# Multi-technique characterization of rhodium gem-dicarbonyls on TiO_2_(110)

**DOI:** 10.1039/d5sc04889c

**Published:** 2025-10-16

**Authors:** Moritz Eder, Faith J. Lewis, Johanna I. Hütner, Panukorn Sombut, Maosheng Hao, David Rath, Paul Ryan, Jan Balajka, Margareta Wagner, Matthias Meier, Cesare Franchini, Gianfranco Pacchioni, Ulrike Diebold, Michael Schmid, Florian Libisch, Jiři Pavelec, Gareth S. Parkinson

**Affiliations:** a Institute of Applied Physics, TU Wien Vienna Austria pavelec@iap.tuwien.ac.at eder@iap.tuwien.ac.at; b Institute of Theoretical Physics, TU Wien Vienna Austria; c Faculty of Physics and Center for Computational Materials Science, University of Vienna Vienna Austria; d Dipartimento di Fisica e Astronomia, Università di Bologna Bologna Italy; e Department of Materials Science, University of Milano-Bicocca Milano Italy

## Abstract

Gem-dicarbonyls of transition metals supported on metal (oxide) surfaces are common intermediates in heterogeneous catalysis. While infrared (IR) spectroscopy is a standard tool for detecting these species on powder catalysts, the ill-defined crystallographic environment renders data interpretation challenging. In this work, we apply a multi-technique surface science approach to investigate rhodium gem-dicarbonyls on a single-crystalline rutile TiO_2_(110) surface. We combine spectroscopy, scanning probe microscopy, and density functional theory (DFT) to determine their location and coordination on the surface. IR spectroscopy shows the successful creation of gem-dicarbonyls on a titania single crystal by exposing deposited Rh atoms to CO gas, followed by annealing to 200–250 K. Low-temperature scanning tunneling microscopy (STM) and non-contact atomic force microscopy (nc-AFM) data reveal that these complexes are mostly aligned along the [001] crystallographic direction, corroborating theoretical predictions. Notably, X-ray photoelectron spectroscopy (XPS) data reveal multiple rhodium species on the surface, even when the IR spectra show only the signature of rhodium gem-dicarbonyls. As such, our results highlight the complex behavior of carbonyls on metal oxide surfaces, and demonstrate the necessity of multi-technique approaches for the adequate characterization of single-atom catalysts.

## Abstract 2

The study investigates rhodium gem-dicarbonyls on titania with a multi-technique approach, using XPS, IR, DFT+*U*, and scanning probe microscopy. The findings have important implications for the rapidly growing field of single atom catalysis.

## Graphical abstract

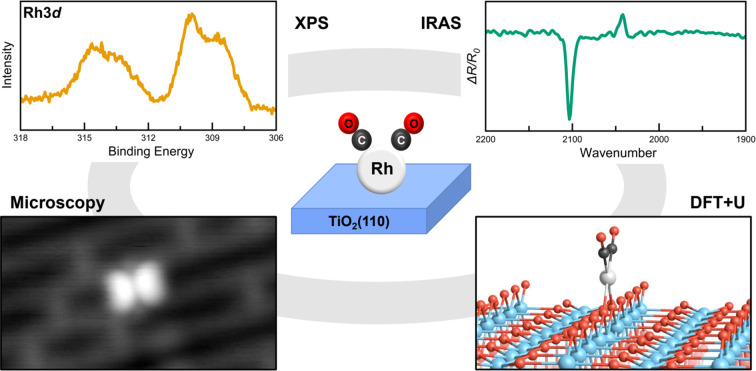

## 1 Introduction

Transition metal carbonyl compounds, including those of Rh, Pd, and Pt, play a pivotal role in both homogeneous and heterogeneous catalysis.^1^ Their utility arises from the strength of the metal–CO bond, which is strong enough to stabilize the compound yet weak enough to render CO an exchangeable ligand. As a result, transition metal carbonyls are established catalysts for a number of processes, such as acetic acid synthesis, hydroformylation, Reppe chemistry, and polymerization reactions.^2^ Metal carbonyls are also integral to the field of single-atom catalysis (SAC). An isolated single atom is typically prone to sintering when it lacks sufficient coordination on the surface, but can be stabilized by coordinating to suitable ligands. Transition metal carbonyls are therefore a direct link between homogeneous and heterogeneous SAC, since the single atom anchored to the surface resembles a metal complex with (exchangeable) ligands.^3–6^ Hence, transition metal carbonyls are garnering significant attention as precursors or intermediates in SAC systems.^7^ For noble metals like Pt, Ir, Pd, and Rh, single-atom dicarbonyls are frequently observed on supports such as zeolites, alumina, ceria, and titania.^7–9^ These species, often referred to as geminal (gem-)dicarbonyls, have been extensively studied, particularly in the case of Rh due to its relevance in, *e.g.*, catalytic converters or the hydroformylation reaction.^10–12^ IR spectroscopy has been the primary technique for detecting gem-dicarbonyls, which yield two characteristic peaks originating from the symmetric and asymmetric vibrational modes of the CO molecules. For Rh, these are typically located between 2120–2075 cm^−1^ and 2053–1989 cm^−1^, respectively.^9^ Recently, Rh carbonyl species have once more received considerable attention as surface species involved in catalytic reactions.^13–19^ Christopher and co-workers have expanded the scope by experimental and computational studies of Rh gem-dicarbonyls on various metal oxide supports for applied catalysis.^17,20,21^ Their works elucidate the creation and stability of Rh carbonyls under various conditions. This includes a study of different Rh carbonyls on rutile TiO_2_ that form after activation of the catalyst by heating in CO. Complementary DFT calculations by Sautet and co-workers shed light on their relative stability as a function of the chemical potentials of O_2_ and CO.^16^ This particular work demonstrated how Rh adatoms adapt to the conditions dictated by the environment, and that the gem-dicarbonyl is the dominant form under reducing conditions in the presence of CO. Despite these advancements in understanding transition metal carbonyls on oxidic supports, comprehensive atomic-level studies using diverse experimental techniques remain scarce. IR spectroscopy remains the main tool for their investigation on powders; however, the IR spectral bands for gem-dicarbonyls in the literature span a range of approximately 50 cm^−1^, and band intensities can be unreliable as quantitative measures.^9^ The transition dipole moment of a molecule determines its signal intensity and can differ vastly depending on the adsorption site and the interplay of donation and back-donation of electron density. This effect is especially pronounced for CO, making the use of additional analytic techniques particularly important for characterizing carbonyl species.^7,9,22^ Moreover, conventional DFT methods to determine the calculated frequencies of the IR absorption bands are often inaccurate.^23,24^ To address these challenges, this study employs a multi-technique approach on an idealized model system. Using a rutile TiO_2_(110) single crystal in ultra-high vacuum (UHV), we leverage a newly developed infrared reflection absorption spectroscopy (IRAS) setup,^25^ linking our findings to those in the literature with IR spectroscopy as a bridging technique. Complementary low-temperature STM and nc-AFM provide direct visualization of Rh gem-dicarbonyls on the surface, pinpointing their location and coordination on TiO_2_(110). Additionally, XPS data provide information about the chemical state of Rh on the surface. DFT calculations rationalize the experimental findings from IRAS and XPS. This comprehensive approach establishes a benchmark for Rh gem-dicarbonyls and underscores the potential pitfalls of relying solely on a single technique for characterizing (single-atom) surface species.

## 2 Results

### 2.1 IRAS spectra of Rh gem-dicarbonyls

#### 2.1.1 Experimental IRAS frequencies

**Fig. 1:**
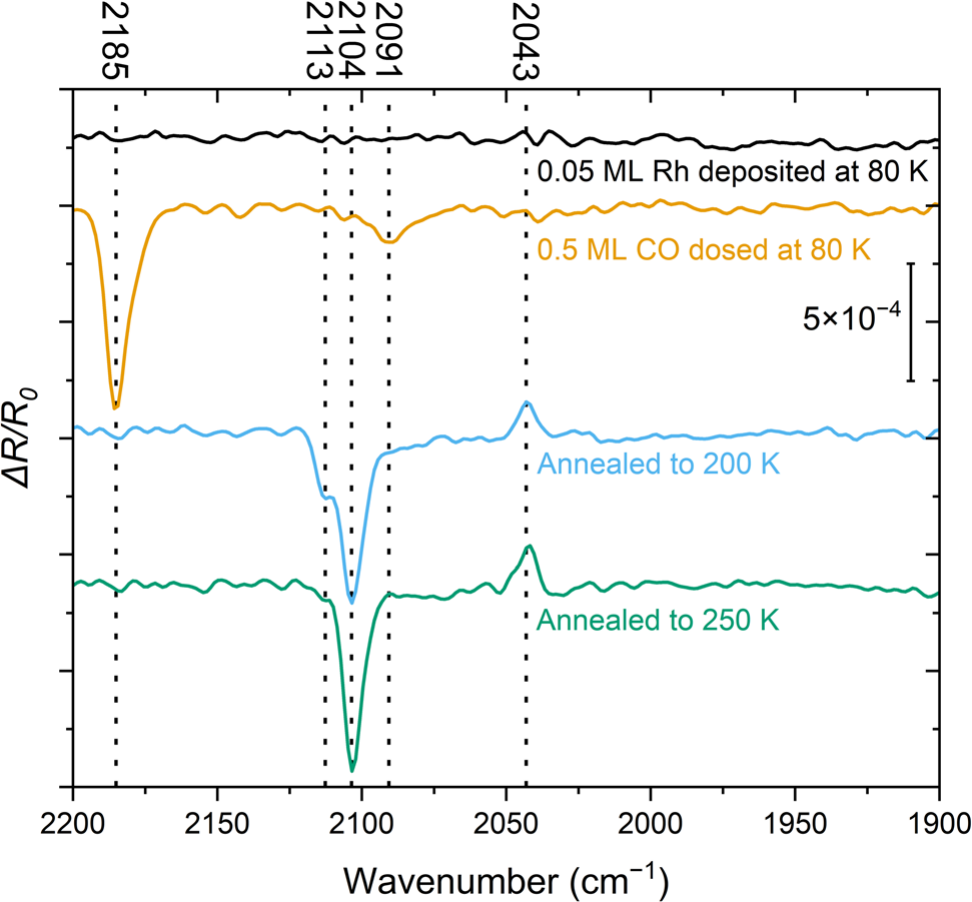
IRAS spectra (baseline-corrected, obtained with p-polarized light, [001] direction in the incidence plane, 4 cm^−1^ resolution, 60 kHz mirror velocity, 4000 scans recorded over ≈10 min at 80 K for each spectrum) of 0.05 ML Rh deposited at 80 K on TiO_2_(110) (black trace), with additional 0.5 ML CO dosed at 80 K (orange trace), after heating to 200 K (blue trace), and after heating to 250 K (green trace). The peaks at 2104 cm^−1^ and 2043 cm^−1^ are assigned to the symmetric and asymmetric vibrational modes of the Rh gem-dicarbonyl, respectively. The reference spectrum was recorded before the series and used for all subsequent measurements.

Two methods for synthesizing Rh gem-dicarbonyls on TiO_2_(110) in UHV are reported in the literature, either by decomposing Rh clusters under high pressures of CO gas,^26^ or by decomposing Rh(CO)_2_Cl_2_ complexes.^27^ However, the first approach was not possible in a clean fashion with our experimental setups, and the latter yields significant amounts of Cl on the surface, which we sought to avoid due to its potential impact on the electronic and geometric structures of the system.^28^ We have shown previously that single Rh atoms can be immobilized on TiO_2_(110) by evaporating Rh onto the surface at 80 K,^29^ and we used this method as a basis to synthesize the Rh gem-dicarbonyls as illustrated in the following. Fig. 1 shows IRAS spectra of 0.05 ML Rh on TiO_2_(110) deposited at 80 K before and after CO adsorption. The spectra were recorded sequentially at 80 K. The accompanying annotations describe the specific sample treatments applied prior to each measurement.

The spectrum recorded immediately after Rh deposition at 80 K (Fig. 1, black) does not show any detectable features in the relevant wavenumber range of 2200–1900 cm^−1^ where the CO-stretch vibration of transition metal carbonyls is commonly found. This indicates that CO adsorption from the residual gas in the UHV chamber is negligible. After adding 0.5 ML CO at 80 K (Fig. 1, orange), we identify two signals: an intense peak at 2185 cm^−1^, attributed to CO on TiO_2_(110),^25,30^ and a band around 2091 cm^−1^. The latter falls within a region where multiple species of Rh carbonyls have been postulated on metal oxides and zeolites.^9,31,32^ The rather broad peak suggests a mixture of species. After flashing the sample to 200 K and cooling it back to 80 K (Fig. 1, blue), the CO/TiO_2_(110) peak has disappeared, consistent with the desorption of CO from this surface at ≈150 K.^30^ Furthermore, the feature at 2091 cm^−1^ is no longer present. Instead, we observe three species at 2113 cm^−1^, 2104 cm^−1^, and 2043 cm^−1^. The latter two are characteristic of the Rh gem-dicarbonyl^27^ and align with the expected parallel and perpendicular contributions of p-polarized light over the measured angular range.^25^ The azimuthal orientation of the gem-dicarbonyls on single-crystalline metal oxides can therefore be inferred from the IR data, since the surface selection rule that governs reflectivity on metallic surfaces does not apply to dielectric sub-strates.^25,27,30,33,34^ In our setup, the principal ray of the IR beam projected onto the surface is parallel to [001], *i.e.*, parallel to the Ti and O rows. The azimuthal orientation of the gem-dicarbonyls on single-crystalline metal oxides can be inferred from the appearance of the positive Δ*R*/*R*_0_ peak; this peak would not appear in p-polarization for asymmetric stretch in the [11̄0] direction (an azimuthal orientation of the gem-dicarbonyl perpendicular to the Ti and O rows). When Rh dicarbonyls are measured on powders whose surfaces are randomly oriented, these two peaks are observed as two minima of the reflectance (maxima of absorbance) in IR spectra. However, our IR beam is reflected on a single crystal surface at a well-defined range of incidence angles, chosen such that the symmetric (2104 cm^−1^) and asymmetric stretch (2043 cm^−1^) result in a lower and higher reflectivity, respectively. This results from the orientation of the dipole moments and the Fresnel equations, as confirmed by simulations.^25^ Following annealing to 250 K (Fig. 1, green trace) the 2113 cm^−1^ feature disappears, while the other two signals associated with the gem-dicarbonyl remain. We cannot assign the peak at 2113 cm^−1^ to one certain species at this point, but we attribute it to a metastable Rh-carbonyl species.^9,31^ By annealing to room temperature and above (Fig. S1), the Rh dicarbonyls disappear and a band around 2020–2040 cm^−1^ emerges, where CO on metallic Rh is typically located.^35^ This is clear evidence that the previously observed Rh species have agglomerated to form CO-covered Rh clusters. While we have observed these vibrational frequencies of the carbonyl species most frequently in our experiments, we have occasionally observed a slight shift of 1–4 cm^−1^ towards higher wavenumbers. We ascribe this to differences in the preparation procedure, and to an enhanced number of adsorbates from the residual gas of the UHV chamber, interacting with the gem-dicarbonyls when the adsorbates are in close proximity to them.

An alternative synthetic route towards the Rh gem-dicarbonyls involves depositing Rh onto a CO-covered TiO_2_(110) surface. This approach has been successfully employed for creating Fe complexes on ligand-covered FeO(111) single crystals.^36,37^ Fig. S2 shows the corresponding IRAS spectra. This method results in the formation of multiple Rh carbonyl species, even after annealing. It hence appears less effective for the selective synthesis of a single type of Rh gem-dicarbonyl on TiO_2_(110).

#### 2.1.2 DFT calculations on Rh gem-dicarbonyls

**Fig. 2:**
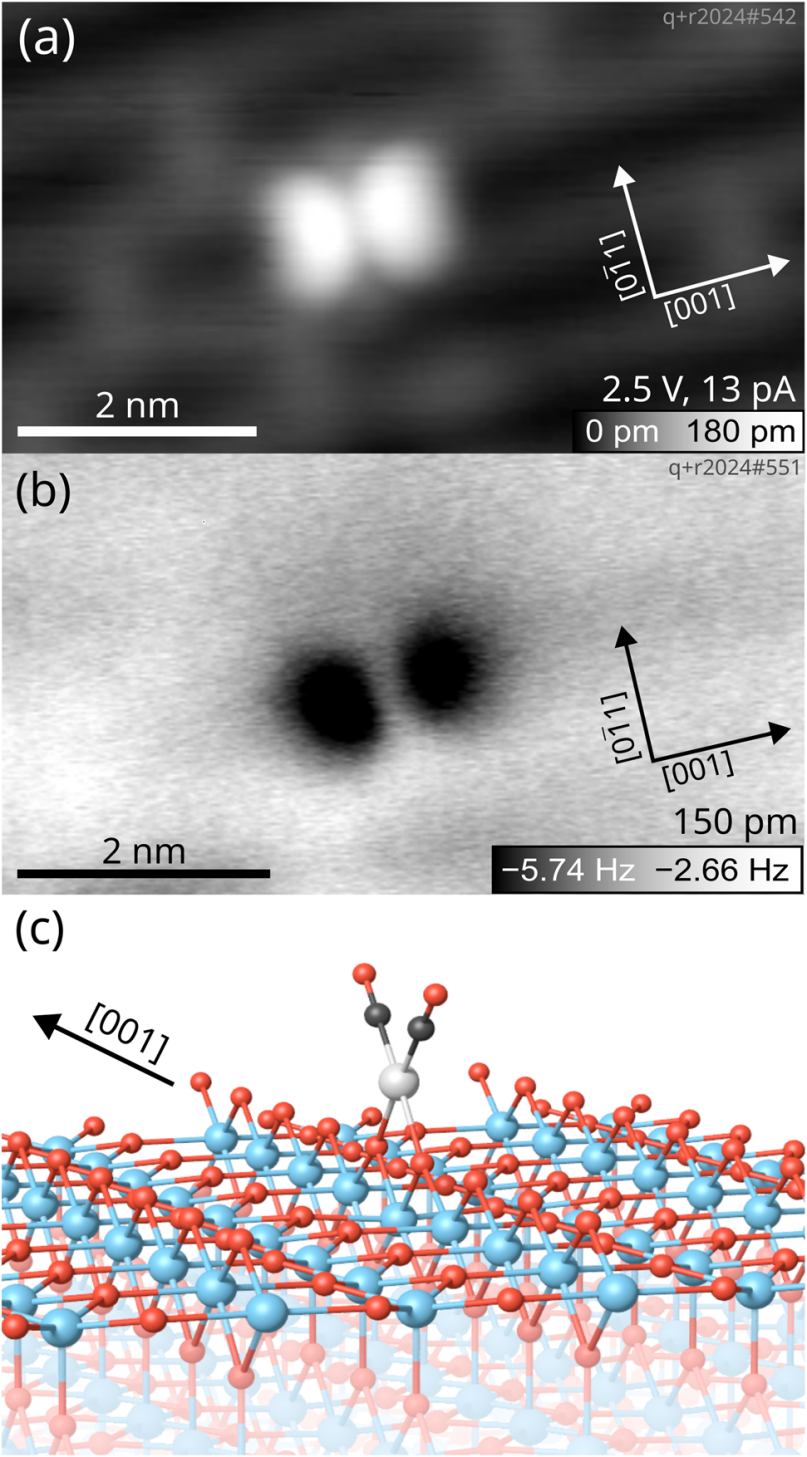
Low-temperature scanning probe images of a Rh gem-dicarbonyl on TiO_2_(110) taken at 14 K with a Cu-terminated tip. (a) Empty-states STM image showing the two CO molecules of the Rh gem-dicarbonyl appearing as bright lobes on the TiO_2_(110) surface. (b) Constant-height nc-AFM image of the same gem-dicarbonyl with a metallic tip, imaging the two CO molecules as dark blobs due to the attractive interaction between the metal tip and the O atoms of the CO (amplitude = 150 pm, *U* = 0.0 V). (c) The optimized structure of the Rh gem-dicarbonyl on the TiO_2_(110) surface obtained by DFT+*U* calculations (Ti: blue; O: red; C: black; Rh: grey).

The optimized ground-state structure of the Rh gem-dicarbonyl was determined using DFT+*U* calculations. Fig. 2c illustrates its arrangement on the TiO_2_(110) surface: the Rh atom (light grey) is positioned between two bridge-bonded O anions (red). The two CO molecules (with C in black) attached to the Rh atom are aligned parallel to the [001] direction. This is the lowest-energy Rh gem-dicarbonyl configuration found in our calculations and in an earlier work by Sautet and co-workers.^16^ The adsorption energy per CO molecule in the square-planar geometry is energetically more favorable by nearly 0.65 eV compared to the tetrahedral alternative, in which the CO molecules are aligned perpendicular to the O and Ti rows (Fig. S3). The calculated Bader charge of the Rh in the gem-dicarbonyl of Fig. 2c is +0.68*e*, which is typical for a Rh^1+^ state. This means that one Ti atom in the subsurface layer is reduced from Ti^4+^ to Ti^3+^.^29,38^ These findings align with the well-known preference of Rh^+^ complexes for square-planar over tetrahedral geometries, as observed in various coordination compounds.^39^

Our calculated CO vibrational frequencies are 2098.9 cm^−1^ and 2042.6 cm^−1^ at the HSE06 level (2080 cm^−1^ and 2021 cm^−1^ with the optPBE-vdW functional). These values agree with our experimental observations within the accuracy of DFT expected for such calculations, particularly regarding the difference between symmetric and asymmetric stretching modes (61 cm^−1^ in the experiment and 56 and 59 cm^−1^ in the HSE06 and optPBE-vdW calculations, respectively). With optPBE-vdW, the difference between the two modes exhibits a smaller error than their individual absolute frequency values due to error compensation from the shared approximation. We considered the possibility of Rh monocarbonyl species, but our calculated CO vibrational frequency with optPBE-vdW for a Rh monocarbonyl (Fig. S3) is 2011 cm^−1^, which is not observed in our experimental IR spectra. Therefore, we ascribe the IR peaks at 2104 cm^−1^ and 2043 cm^−1^ to the symmetric and asymmetric stretch of Rh^+^(CO)_2_, respectively.

### 2.2 Rh gem-dicarbonyls in low-temperature scanning probe microscopy

To further confirm the conformation of Rh gem-dicarbonyls, we employed low-temperature STM and nc-AFM. Fig. 2 presents microscopy images taken at a sample temperature of 14 K. The sample was prepared by depositing 0.05 ML Rh onto the TiO_2_(110) surface, followed by dosing 1 langmuir (L) of CO and annealing to ≈270 K. An overview scan is presented in Fig. S4.

The overview scan in Fig. S4 shows the presence of clusters apart from the features we ascribe to the gem-dicarbonyls (green circles). We believe that the abundance of clusters is due to the fact that the annealing temperature in the STM experiment (≈270 K) was higher than in the IR experiment (250 K). However, it is plausible that clusters are present on the sample used in the IR experiment as well, but are not detected if they have no CO molecules adsorbed. In the empty-states STM image in Fig. 2a, we observe the characteristic features of the TiO_2_(110) surface.^40^ The grey and black rows along the [001] direction correspond to the Ti cations and bridge-bonded O anions, respectively. Grey bridges across the black rows are O vacancies,^41^ evidence for a reduced, non-stoichiometric surface. We find a distinct feature in the form of a bright double-lobe oriented along the [001] direction, located on top of a bridge-bonded O row. The center of the lobes is exactly located between two bridging oxygen anions, as illustrated by the unit cell grid centered on the oxygen vacancies (see Fig. S5). The bright double lobe stems from the two CO molecules aligned in a plane parallel to the Ti and O rows, in excellent agreement with our spectroscopic and computational results, and calculations by Sautet and co-workers.^16^ A nc-AFM image of the same spot on the surface in Fig. 2b corroborates this interpretation. We find again two lobes aligned in the [001] direction, which appear dark in this case due to the attractive interaction between the metallic Cu-terminated tip and the O of the CO molecules.

**Fig. 3:**
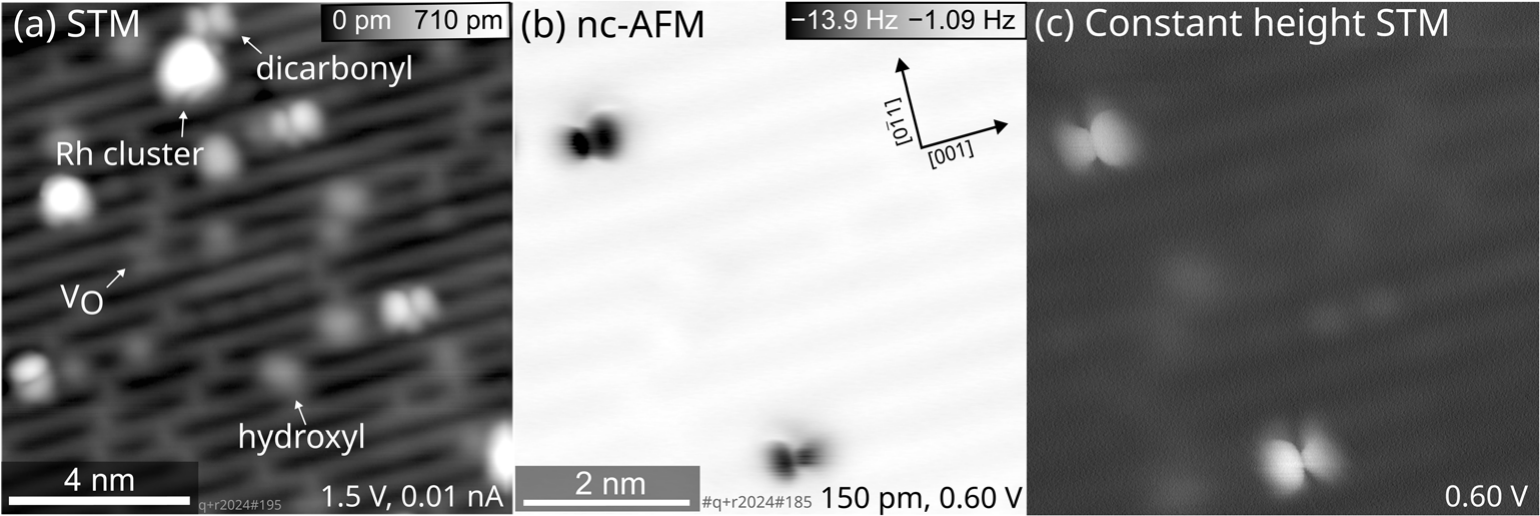
Low-temperature SPM images of Rh gem-dicarbonyls on TiO_2_(110) acquired at 14 K with a Cu-terminated tip. 0.005 ML Rh were deposited at 100 K, exposed to 1 langmuir (L) (corresponding to ≈0.88 ML) CO at 100 K, and heated to 270 K. (a) Empty-states STM image of a 15 nm × 15 nm area. (b) Constant-height nc-AFM image of a region of (a) containing two gem-dicarbonyls with a metallic tip, imaging the two CO molecules as black spots due to their attractive interaction to the tip (amplitude = 150 pm). (c) Constant-height STM image of the same area acquired at the same time as the nc-AFM scan.

As mentioned above, the STM images show a substantial number of species attributed to Rh_*n*_ clusters at a Rh coverage of 0.05 ML and successive CO adsorption and annealing to ≈270 K. By using the same preparation of Rh gem-dicarbonyls on the TiO_2_(110) surface but aiming at a lower Rh coverage (0.005 ML, *i.e.*, one tenth of the previous coverage), the number of large clusters is reduced, but Rh clusters cannot be fully avoided. Fig. 3 shows SPM images of the surface after this preparation.

An overview scan covering a larger surface area (Fig. 3a) revealed Rh clusters as well as double-lobed, inequivalent features apart from the typical surface species like hydroxyls and O vacancies. A constant-height image of a region of Fig. 3a shows an asymmetry of two gem-dicarbonyls, depicted in the frequency shift in Fig. 3b and the simultaneously recorded tunneling current in Fig. 3c. Under all imaging conditions, this asymmetry of the individual double-lobed features stays consistent. The opposite orientations of these inequivalent CO molecules suggest that the effect is not an artifact from a slightly asymmetric tip. Instead, the inequivalence could arise from distinct interactions between the two CO molecules of the dicarbonyl and the TiO_2_(110) surface, causing a tilt of the structure in the [001] or [001̄] direction. One plausible explanation involves interaction with nearby OH groups, which are ubiquitous on TiO_2_(110) surfaces. Our DFT calculations show that the interaction between one Rh-bound CO and a hydroxyl group on the O rows would indeed lead to an asymmetric position of the CO molecules with respect to the Rh atom (Fig. S6). Recent calculations for anatase TiO_2_(001) by Christopher and Pacchioni lend support to this hypothesis, demonstrating that OH groups can interact with CO molecules, influencing their geometry and alignment.^17^ We therefore assign the different size of the lobes in nc-AFM to a neighboring OH group next to one CO molecule of the Rh gem-dicarbonyl complex.

### 2.3 XPS spectra of Rh and C

**Fig. 4:**
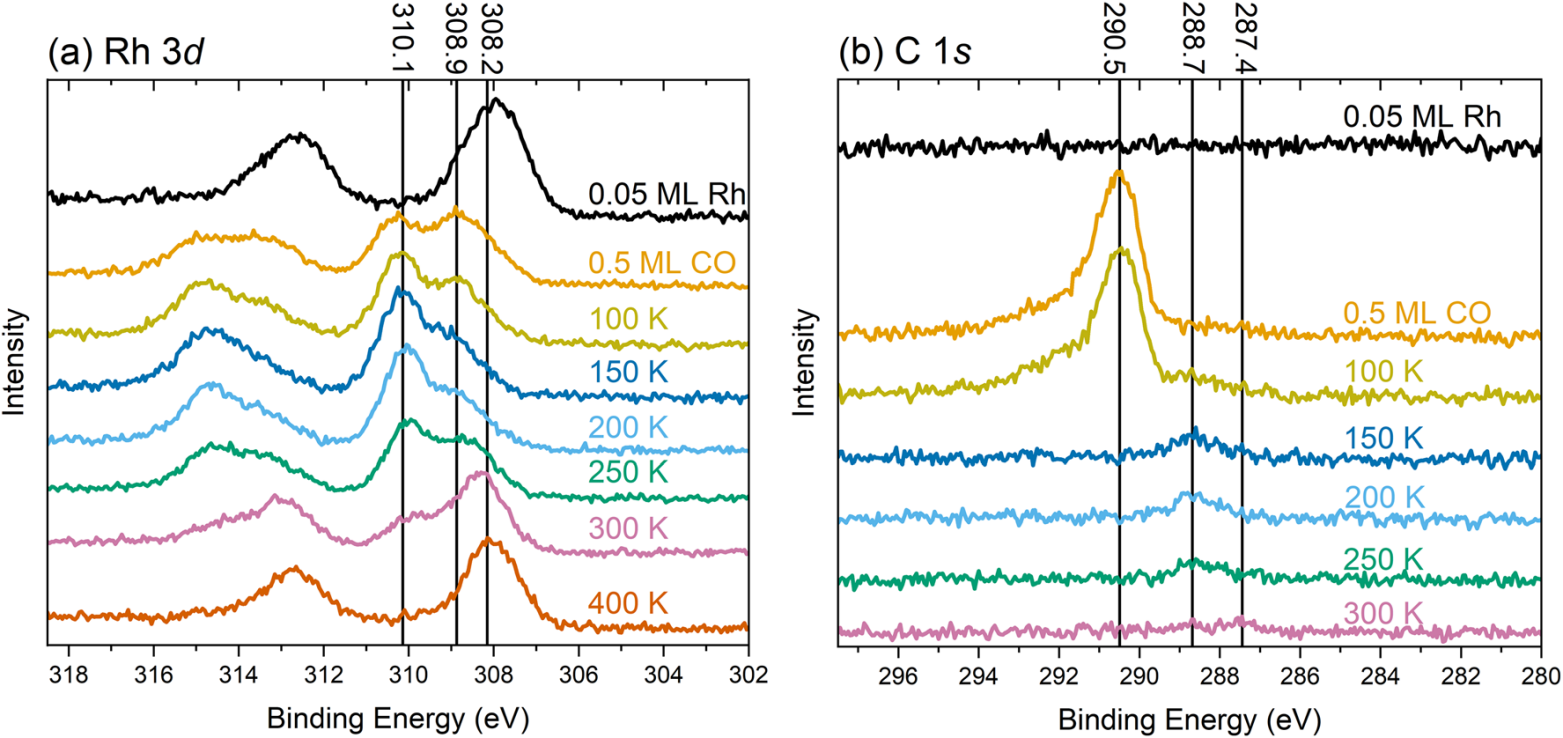
XPS spectra of (a) the Rh 3d and (b) C 1s regions taken at 80 K using monochromatized Al K_α_ radiation at 70° grazing emission for enhanced surface sensitivity. The colored text corresponding to each spectrum indicates the treatment before the measurement.

To understand the chemical nature of the Rh species, we recorded XPS spectra during the same experiment as for the IRAS measurements depicted in Fig. 1. Fig. 4 presents XPS spectra of the Rh 3d and C 1s region of the TiO_2_(110) surface at 80 K after various treatments, as indicated in the corresponding annotations.

The Rh 3d spectrum in Fig. 4a shows the expected doublet after depositing 0.05 ML Rh at 80 K (black trace). The Rh 3d_5/2_ peak is centered around 308 eV, consistent with a prior study of this system.^29^ The width of the peak suggests the coexistence of different species, presumably the single atoms and very small clusters as observed by STM/nc-AFM in Fig. S7. Due to the broadness of the peak, the presence of metallic Rh, centered around 307.7 eV (Fig. S8) seems likely. Adding 0.5 ML CO at 80 K (Fig. 4a, orange trace), splits the Rh 3d_5/2_ peak into two maxima of comparable intensity at 310.1 eV and 308.9 eV. Again, the broadness of both peaks, in particular the tail below 308.9 eV, suggests contributions from different chemical surroundings. Upon heating to 100 K, 150 K, and 200 K (yellow, dark blue, and light blue traces, respectively), we still observe the two species at the same position, although their relative intensities change. At these temperatures, the higher-binding-energy component dominates. At 250 K (green trace), the intensity shifts slightly in favor of the lower-binding-energy component, and a shoulder emerges at 308.2 eV, indicative of a larger, agglomerated species. The observation of the component around 308.2 eV is consistent with the observation of clusters in STM in Fig. S4. After annealing to 300 K (pink trace in Fig. 4a), the species at 308.2 eV dominates, with diminished intensities for the two components at higher binding energy. The substantial reduction in the intensity of the peak at 310.1 eV coincides with the loss of the bands from the gem-dicarbonyl in IRAS. Thus, we assign the peak at 310.1 eV to the Rh gem-dicarbonyl, and the species around 308.9 eV to clustered Rh-carbonyl species. This observation aligns well with a study by Hayden *et al.*,^42^ who observed the agglomeration of Rh gem-dicarbonyls on TiO_2_(110) at room temperature; this was attributed to the impact of hydrogen (*i.e.*, hydroxyl groups) on the surface. Our result also aligns with observations by Frederick *et al.*, who found a similar Rh 3d binding energy (310.2 eV) for Rh gem-dicarbonyls on alumina.^43^ The lower binding energy component at 308.9 eV likely originates from the carbonylation of small Rh clusters that formed upon deposition, as depicted in Fig. S7. The variety of Rh species in Fig. 4a is much larger after annealing to 300 K compared to depositing Rh directly at 300 K (see Fig. S8). XPS of the latter yields only one significant Rh 3d_5/2_ peak at a neutral Rh position of 307.7 eV, originating from clusters as detected in STM.^29^ After heating to 400 K (red in Fig. 4a), we find one pronounced species around 308 eV, close to the initial position after Rh deposition in the absence of CO. This peak is assigned to Rh clusters with a more metallic character. DFT core-level calculations using the initial-state approximation reveal that the binding energies of Rh 3d for a Rh monocarbonyl and a Rh gem-dicarbonyl are shifted to higher values by 0.79 and 1.46 eV, respectively, relative to the Rh single-atomic ground-state configuration on TiO_2_(110). The Rh 3d_5/2_ peak position around 310 eV attributed to the gem-dicarbonyl could be taken as representative of a Rh^3+^ charge state. However, the Bader charge analysis and the number of reduced Ti atoms indicate that this Rh species is in the Rh^+^ state. Hence, the shift of the Rh peaks towards higher binding energies upon CO exposure is due to intramolecular redistribution of electron density, but not due to a change in formal charge state. This is in line with other single-atomic carbonyls on Fe_3_O_4_(001).^44^ Our DFT results (Fig. S9) show that increasing the Rh coordination by binding to the CO molecules in the square-planar configuration shifts the energies of d-band states to lower values compared to adsorbed, bare Rh. This also suggests that the available mobile charge around the Fermi edge to screen out the core hole is substantially reduced at the Rh site. In addition to initial-state effects such as an overall downshift of the Rh states, this could also lead to final state effects. The C 1s spectra in Fig. 4b exhibit no detectable signal for the Rh-decorated surface (black trace). Following CO dosing (orange trace), a peak emerges at 290.5 eV, which we ascribe to CO adsorbed on TiO_2_. Heating to 100 K (yellow trace) induces a shoulder at 288.7 eV, which we ascribe to CO on Rh. This corroborates evidence from IRAS (Fig. 1, orange trace) which suggests that CO is immobile after landing on the surface at 80 K. Hence, it requires thermal energy to facilitate CO diffusion, and to lift a Rh atom onto the bridging O rows (see Fig. S9a to S9b) to form Rh gem-dicarbonyls (Fig. S9c). After annealing to 150 K (Fig. 4b dark blue trace), the peak of CO on TiO_2_ (290.5 eV) has vanished due to CO desorption from the substrate. This agrees again with the corresponding result in the IR spectra in Fig. 1 (blue trace) and the literature.^30^ When increasing the temperature to 200 K and 250 K (light blue and green trace in Fig. 4b, respectively), the CO/Rh peak at 288.7 eV remains stable in position and intensity. At 300 K, we find its intensity diminished, due to the desorption of the CO from the Rh gem-dicarbonyls. This coincides with the emergence of a weak peak around 287.4 eV. This new signal is attributed to CO adsorbed on Rh clusters, reflecting the aggregation of Rh species as observed in the Rh 3d spectra.

## 3 Discussion

The infrared spectra (Fig. 1) demonstrate the facile synthesis of Rh gem-dicarbonyls on TiO_2_(110) in UHV, a model system for metal carbonyls anchored to metal oxides.^9,45^ Our synthetic approach avoids high pressures and Cl contaminations on the surface (in contrast to previous works),^27,46^ and is presumably transferable to other transition metals like Ir, Ni, Pt, or Pd, known for forming carbonyl complexes on metal oxide surfaces.^8,9,45^ Our study provides the first low-temperature scanning probe images of Rh gem-dicarbonyls on TiO_2_(110) (Fig. 2). The double-lobed features, aligned along the [001] direction, strongly support a square-planar geometry. This finding contrasts with earlier studies by Hayden *et al.*, which provide evidence for a perpendicular alignment of the Rh gem-dicarbonyls to the Ti and O rows on TiO_2_(110) after dosing [Rh(CO)_2_(Cl)_2_].^33^ However, our spectroscopic results in Fig. 1 are consistent with our computational results and recent work by Tang *et al.*, which predict the alignment along the bridging O rows on TiO_2_(110),^16^ as well as our SPM images. The square-planar geometry arises from the coordination of Rh with two CO ligands parallel to the Ti and O rows, reflecting the intrinsic preference of Rh^+^ complexes for square-planar over tetrahedral conformations.^39^ We have observed distortions of the dicarbonyls (Fig. 3), possibly due to the interaction of CO ligands with surface OH groups, as suggested by DFT calculations (Fig. S6). These distortions could influence the catalytic activity and selectivity of gem-dicarbonyl species.^47^ Our XPS data (Fig. 4) provide critical insights into the electronic states of Rh species. Note that the Rh 3d peaks after deposition are located at higher binding energies (≈308 eV for Rh 3d_5/2_) compared to neutral, bulk-like Rh (≈307 eV),^48^ likely due to final-state effects of the single adatoms and small clusters.^29^ The broadness of the signal is in excellent agreement with the fact that we find single atoms as well as small clusters in STM after deposition. Exposing the Rh surface species to CO and heating this system creates Rh carbonyls from single atoms and possibly the partial break-up of clusters.^49^ These processes are reflected in changes in the Rh 3d and C 1s spectra already at 80 K (CO exposure) and upon annealing to 100 K. The Rh 3d signal splits into components with lower and higher binding energy (Fig. 4a). The feature with higher binding energy at 310.1 eV is identified as originating from the gem-dicarbonyl, in agreement with the literature.^43^ The 310.1 eV signal increases upon annealing up to 200–250 K. This reflects both enhanced CO diffusion to Rh sites and CO-induced restructuring of Rh species, including jumping onto the rows of 2-fold oxygen (Fig. S9a to S9b) and breaking up of small clusters.^26,49^ This indicates that the Rh gem-dicarbonyls are thermodynamically favorable, but their formation is kinetically hampered at low temperatures. The authors of the work on Rh gem-dicarbonyls on alumina^43^ ascribed the peak at ≈310 eV to a Rh^3+^ species, but our DFT results suggest that the Rh in the gem-dicarbonyl on TiO_2_(110) is in the oxidation state 1+, and we consider a Rh^3+^ state unlikely on the hardly reducible alumina substrate. We also rule out the oxidation state 2+ for the Rh species due the absence of multiplet splitting in the Rh 3d spectra.^50^ The different Rh 3d binding energies with and without CO are rather due to electronic effects attributed to the different coordination, as confirmed by DFT (Fig. S9). The calculated vibration frequencies confirm that the two main peaks observed in the same temperature range as the dicarbonyl signal in XPS (Fig. 1, 2104 cm^−1^ and 2043 cm^−1^) are due to the dicarbonyl. The IRAS shows an additional signal at 2113 cm^−1^ after annealing to 200 K, which disappears upon annealing to 250 K. We note that the disappearance coincides with a slight intensity increase of the gem-dicarbonyl peak at 2104 cm^−1^, but also with an increase of the Rh 3d XPS signal at ≈308 eV (Fig. 4a), which is related to small Rh clusters. Thus, it is unclear whether the species responsible for the 2113 cm^−1^ peak transforms into the “normal” gem-dicarbonyl or creates Rh clusters upon its disappearance.

XPS suggests the presence of small Rh carbonyl clusters with a Rh 3d_5/2_ binding energy around 308.9 eV. It seems surprising that the IRAS spectra do not show any evidence for their presence. However, this is likely due to the heterogeneity of these carbonyl clusters. For clusters with different sizes (different amounts of Rh and CO), the directions of the CO dipole moments and their vibrational frequencies vary substantially.^51^ The resulting signals do not add coherently, and thus no distinct IR feature emerges. It is also possible that some of these Rh carbonyl clusters are overshadowed by the peak of the Rh gem-dicarbonyl asymmetric stretch at 2043 cm^−1^. In any event, upon annealing to temperatures above 250 K, the dicarbonyls vanish and the Rh forms small clusters. These observations are in line with studies proposing the reductive desorption of CO from Rh carbonyls on zeolites,^52^ and the presence of clusters in the STM images in Fig. 3 and S4. The peak intensities of the symmetric and asymmetric stretch in the IRAS spectrum in Fig. 1 are consistent with the calculated IRAS sensitivities for out-of-plane and in-plane vibrations with p-polarized light. According to previous calculations, IRAS is substantially more sensitive to out-of-plane vibrations at incidence angles above 30°.^25^

These results show that relying solely on IR spectroscopy for identifying surface species, for example in single-atom catalysis, can be deceiving. More Rh species can be found in the XPS than one would expect from the IR data showing only a single dicarbonyl species following the 250 K anneal. This is likely due to these IR-invisible species binding to very few or no CO molecules, or the dipole moment of those CO species being too low,^22^ as well as the inhomogeneity not leading to sharp IRAS peaks. Integration of the C 1s peaks in Fig. 4b shows that after CO desorption from the oxide (150 K and higher), the area under the remaining CO signal amounts to about 10% of the initial signal after dosing 0.5 ML CO. However, if all Rh atoms (0.05 ML) were covered by 2 CO molecules, it should amount to 20%. This provides additional evidence that not all Rh species are gem-dicarbonyls, and that some Rh atoms have to be in clusters, as suggested above, based on the lower binding energy component of the Rh 3d signal. The fact that the IR spectroscopy does not detect these significant quantities of clustered species has profound implications for catalysis, especially in single-atom systems, where the catalytic role of single atoms *versus* minor concentrations of clusters is often debated. A multi-technique approach, as demonstrated here, is essential to resolve ambiguities and provide a comprehensive understanding of the active surface species.^53^

## 4 Conclusions

Our results build upon and expand previous findings on gem-dicarbonyls on metal oxide supports. While earlier studies have primarily relied on IR spectroscopy, our multi-technique approach provides a more nuanced characterization of these species. The direct visualization of gem-dicarbonyls *via* STM and nc-AFM bridges the gap between theoretical predictions and experimental evidence. Their stability under realistic operating conditions is limited, with decomposition of the dicarbonyls and formation of Rh clusters occurring at temperatures above 250 K. However, the transition metal gem-dicarbonyls on metal oxides with distinct spectroscopic fingerprints are important intermediates in catalytic processes in general, potentially influencing reaction pathways. Additionally, the suggested interaction with surface OH groups may provide a route for tuning their electronic and geometric properties, opening avenues for site-specific catalysis. The scanning probe images show mainly gem-dicarbonyls oriented along the [001] direction along the Ti and O rows, in agreement with the IRAS data and DFT calculations predicting a fourfold-planar coordination of Rh^+^. The XPS data show that there is more than one Rh species on the surface, attributed to small Rh clusters, invisible in the IR spectra despite being present in considerable amounts and likely covered by CO at these temperatures. This demonstrates the necessity of multi-technique approaches in the investigation of single atoms and carbonyls, and therefore has important implications for single-atom catalysis.

## Author contributions

Moritz Eder: conceptualization, methodology, investigation, original draft, review & editing, project administration. Faith Lewis: investigation, data curation, discussion. Johanna Hütner: investigation, discussion. Jan Balajka: supervision, discussion. David Rath: investigation & discussion. Panukorn Sombut, Maosheng Hao: investigation, formal analysis, discussion. Matthias Meier, Florian Libisch, Cesare Franchini: supervision, discussion. Gianfranco Pacchioni: methodology, discussion. Jiri Pavelec: conceptualization, writing – review & editing, supervision, project administration. Paul Ryan, Margareta Wagner: discussion, validation. Michael Schmid, Ulrike Diebold, Gareth S. Parkinson: validation, resources, writing – review & editing, funding acquisition, discussion.

## Conflicts of interest

There are no conflicts to declare.

## Supplementary Material

SC-OLF-D5SC04889C-s001

## Data Availability

The data that support the findings of this study are available from the corresponding author upon reasonable request. Supplementary information is available. See DOI: https://doi.org/10.1039/d5sc04889c.
