# Supplementary material for: Multi-technique characterization of rhodium gem-dicarbonyls on TiO2(110)
Source: Chem Sci. 2025 Oct 16;16(47):22481–9. doi: 10.1039/d5sc04889c (PMC12551149; doi:10.1039/d5sc04889c)
Supplement: SC-016-D5SC04889C-s001 [file SC-016-D5SC04889C-s001.pdf]

Supplementary Information (SI) for Chemical Science.
This journal is © The Royal Society of Chemistry 2025

Supporting Information for

Multi-Technique Characterization of Rhodium gem-Dicarbonyls on TiO_2(110)

Moritz Eder*,[1] Faith J. Lewis,[1] Johanna I. Hütner,[1] Panukorn Sombut,[1] Maosheng Hao,[2] David Rath,[1] Paul Ryan,[1] Jan Balajka,[1] Margareta Wagner,[1] Matthias Meier,[3] Cesare Franchini,[3,4] Gianfranco Pacchioni,[5] Ulrike Diebold,[1] Michael Schmid,[1] Florian Libisch,[2] Jiri Pavelec*,[1] and Gareth S. Parkinson[1]

[1] Institute of Applied Physics, TU Wien, Vienna, Austria

[2] Institute of Theoretical Physics, TU Wien, Vienna, Austria

[3] Faculty of Physics and Center for Computational Materials Science, University of Vienna, Vienna AT 1090, Austria

[4] Dipartimento di Fisica e Astronomia, Università di Bologna, Bologna IT 40126, Italy

*Corresponding authors: pavelec@iap.tuwien.ac.at, eder@iap.tuwien.ac.at

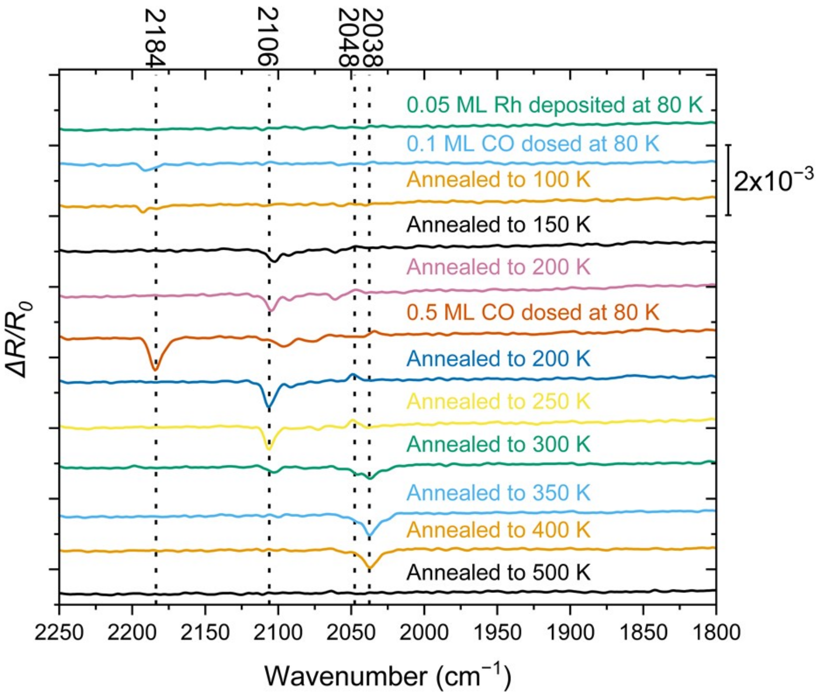

Figure S1. IRAS spectra (baseline-corrected, obtained with p-polarized light, [001] direction in the incidence plane, 4 cm^{-1} resolution, 60 kHz mirror velocity, 4000 scans recorded over ≈10 min at 80 K for each spectrum) of Rh deposited at 80 K on TiO_2(110) (black) with different CO exposures and heat treatments between the measurements as indicated above each curve. The reference spectrum was recorded before the series and used for all subsequent measurements. In contrast to **Figure S2**, Rh is deposited before CO is dosed.

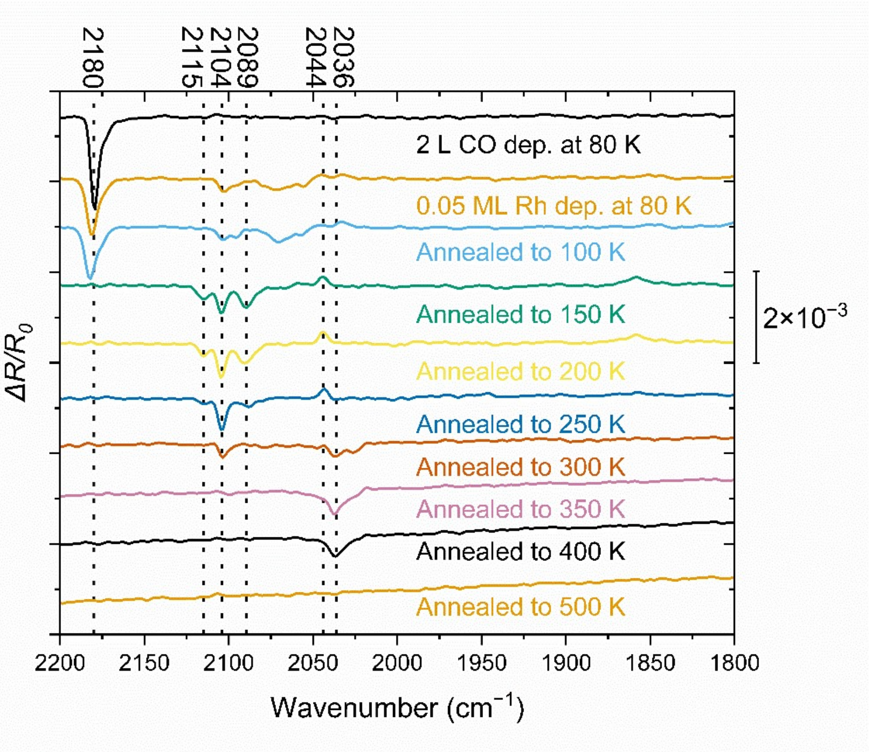

Figure S2. IRAS spectra (baseline-corrected, obtained with p-polarized light, [001] direction in the incidence plane, 4 cm^{-1} resolution, 60 kHz mirror velocity, 4000 scans recorded over ≈10 min at 80 K for each spectrum) of Rh deposited at 80 K onto a CO-covered $TiO_2(110)$ surface, with heat treatments between the measurements as indicated above the curves. 2 L of CO exceed the saturation coverage of CO on $TiO_2(110)$ at 80 K. The reference spectrum was recorded before the series and used for all subsequent measurements. In contrast to **Figure S1**, CO is dosed before Rh is deposited.

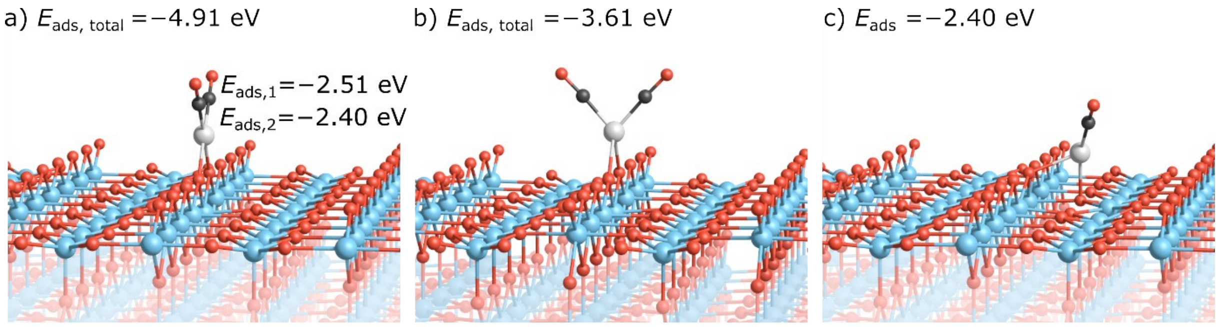

Figure S3. Ball and stick models of Rh carbonyls on TiO_2(110) (blue: Ti; red: O; grey: Rh; black: C. The geometry was determined by means of DFT+U for a) a square-planar conformation, b) a tetrahedral conformation, and c) a monocarbonyl. The total adsorption energy ($E_{ads,total}$) for two CO molecules in the square-planar configuration is energetically more favorable compared to the tetrahedral configuration. The values $E_{ads,1}$ and $E_{ads,2}$ represent the differential adsorption energies for the first and second CO molecules forming the the gem-dicarbonyl, respectively.

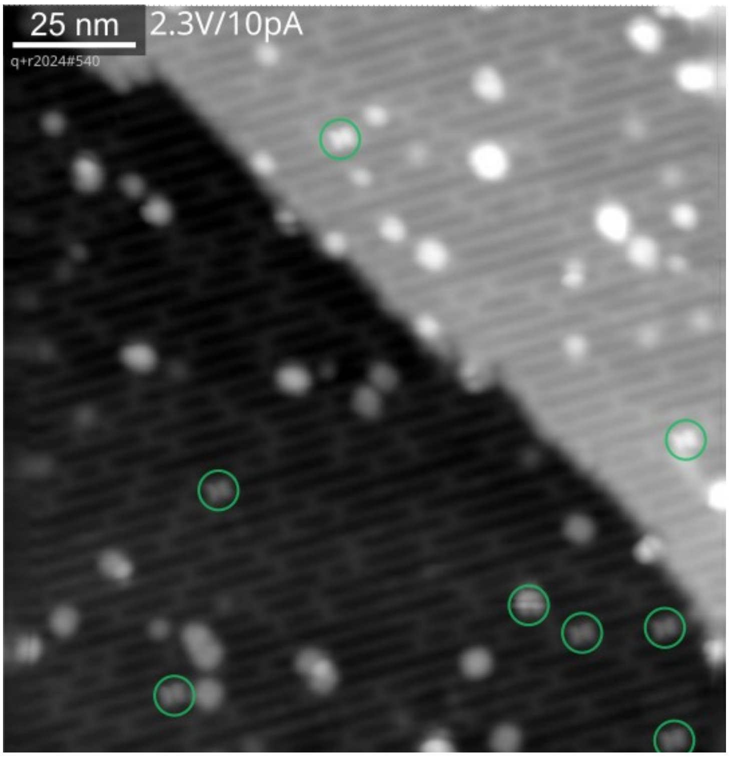

Figure S4. Empty-states STM image (using a Cu-terminated tip, taken at 14 K of 0.05 ML Rh deposited at 100 K on $TiO_2(110)$, subsequent dosing of 1 L CO (corresponding to ≈0.88 ML) at 100 K, and heating to ≈270 K. Note that the temperature here is 20 K higher than in the IRAS experiment (green line in **Figure 1**) and the XPS experiment (green line in **Figure 4**), likely contributing to the agglomerates present in the scanned area. $Rh(CO)_2$ gem-dicarbonyls are marked by green circles.

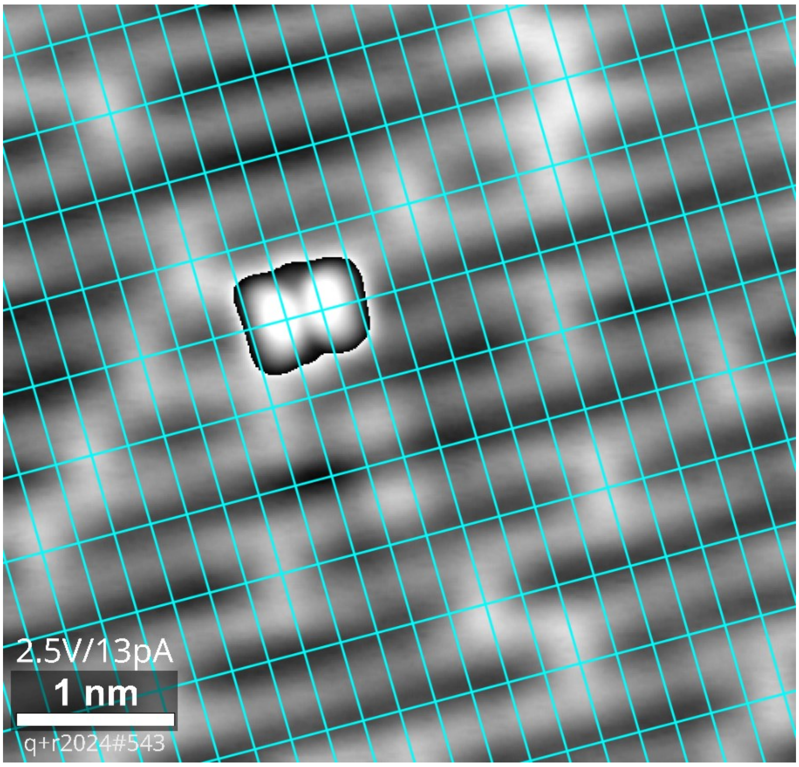

Figure S5. Empty-states STM image (using a Cu-terminated tip) taken at 14 K of 0.05 ML Rh deposited at 100 K on TiO_2(110), after subsequent dosing of 1 L CO (corresponding to ≈0.88 ML) at 100 K, and heating to ≈270 K. The superimposed grid is constructed of rectangles in the size of the surface unit cells, with the nodes of the grid centered on the oxygen vacancies. The center of the bright feature ascribed to the Rh gem-dicarbonyl lies in the middle of two nodes, locating the Rh atom on top the oxygen row in the middle of two oxygen anions, in agreement with the DFT result (**Figure 2c**). Since the apparent height of the dicarbonyl substantially exceeds that of the substrate, a separate grayscale has been used for the highly protruding species.

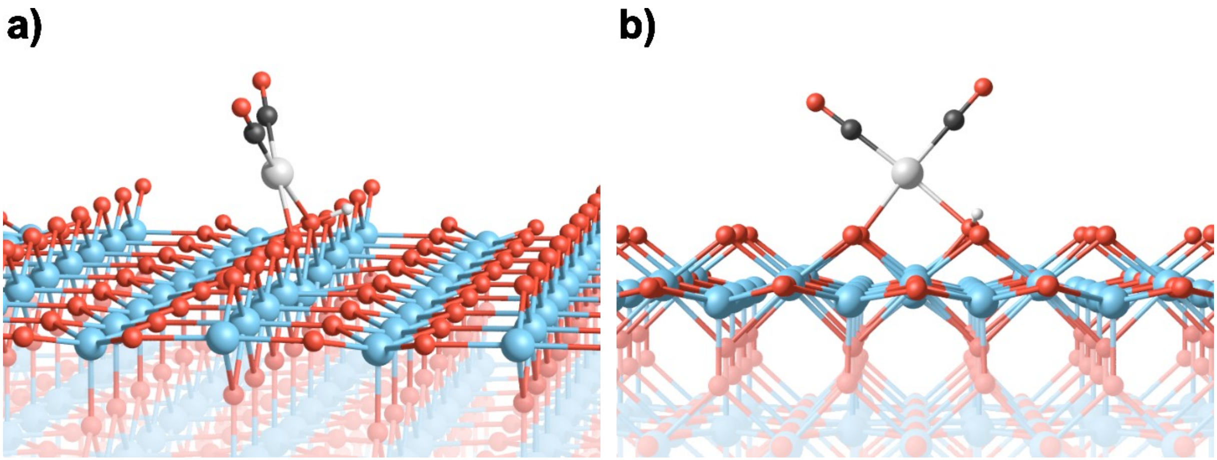

Figure S6. Ball and stick model of a Rh gem-dicarbonyl species on TiO_2(110) in the presence of an additional H atom a) in perspective view and b) in side view. (blue: Ti; red: O; grey: Rh; black: C, white: H) The geometry was determined by means of DFT+U. The pmm symmetry of the undisturbed gem-dicarbonyl (**Figure 2c**) is broken by the interaction with the hydrogen atom forming a hydroxyl group with a bridge-bonded O atom.

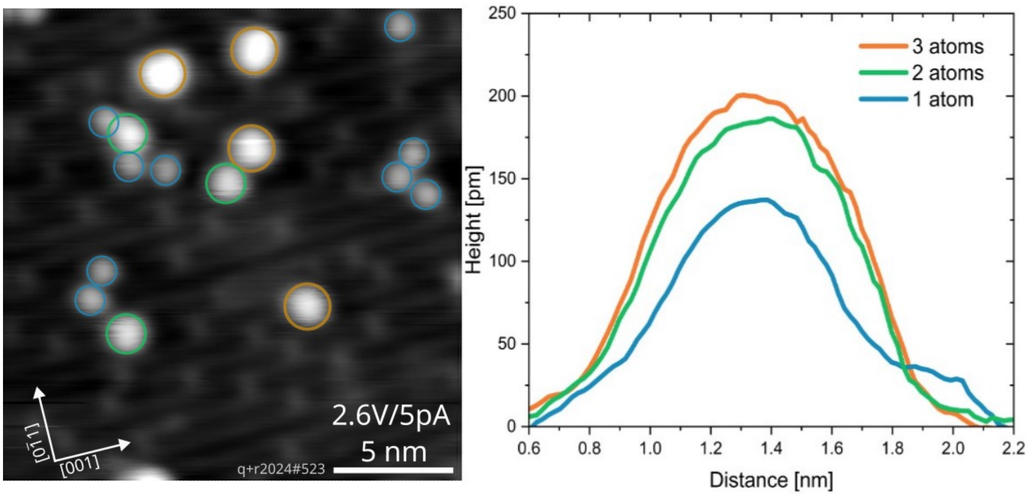

Figure S7. Empty-states STM image taken at 14 K of 0.05 ML Rh deposited at 100 K on TiO_2(110) (left). Profiles of the apparent heights of surface species attributed to Rh single atoms and clusters are shown at the right. The different apparent heights reflected as different brightness of the species in the STM image found after deposition suggest the presence of single Rh atoms (blue) and small clusters (green, orange).

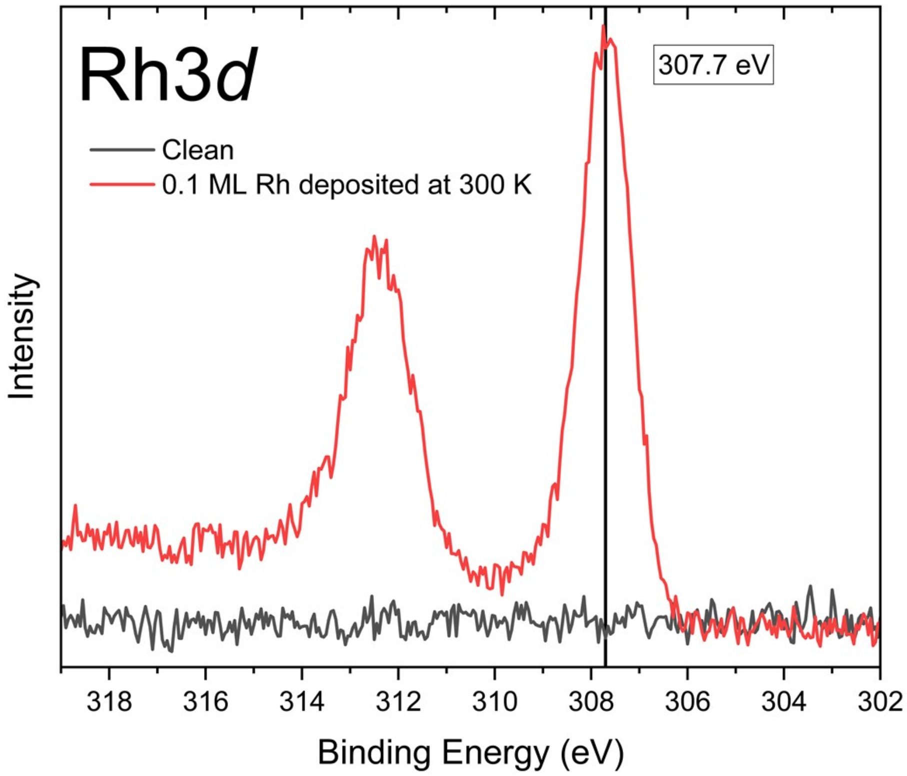

Figure S8. XPS spectra on $TiO_2(110)$ in the Rh3*d* region taken at 300 K using monochromatized Al Kα radiation at 70° grazing emission. The black curve shows a clean $TiO_2(110)$ surface. The red curve shows the same region after Rh (0.1 ML) deposition at 300 K.

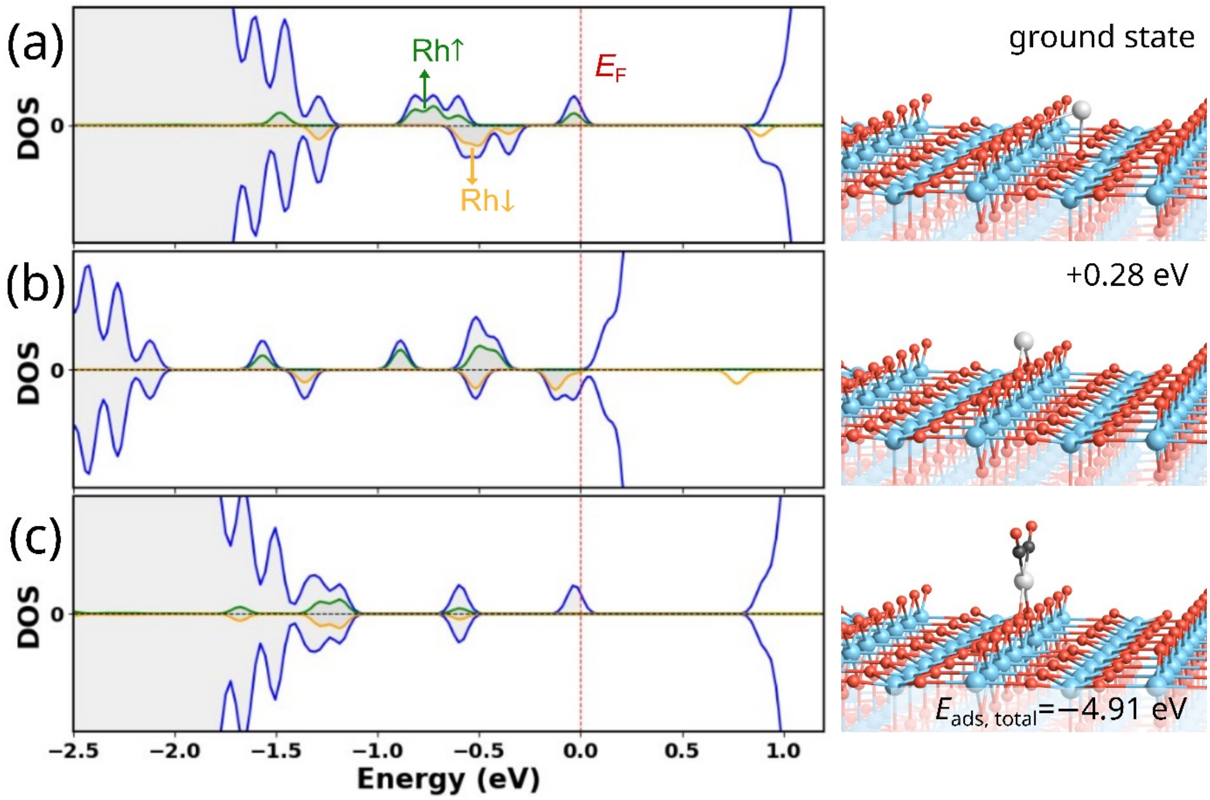

Figure S9. Density of states and corresponding schematic illustrations for a TiO_2(110) surface (Ti: blue, O: red) with one Rh atom (grey) in different configurations: (a) a Rh atom forming an inclined triangular configuration with two oxygen atoms from the first and second layer, (b) a Rh atom above the row of 2-fold oxygen in a bridge configuration, (c) a Rh atom bound to two CO (C: black, O: red) molecules (gem-dicarbonyl), forming a square-planar configuration together with two bridging oxygen atoms. The blue curve represents the total density of states for spin up and spin down, while the green and orange curves show the projected density of states onto the Rh 4*d* orbital for spin up and spin down, respectively. The red dashed line marks the Fermi level. Note that this position assumes an undoped substrate. In experiment, the Fermi level will be close to the valence band minimum due to oxygen deficiency (n-type doping) in all configurations. In the perspective views at the right, energy differences are given with respect to the Rh adatom configuration in (a). Rh binding to a bridging oxygen and a threefold-coordinated oxygen is energetically more favorable than binding to two bridging oxygens by 0.28 eV as shown in (b). Thus, a single Rh adatom in its ground state configuration has to jump from the side of the 2-fold O row onto this row for reaching the preferred gem-dicarbonyl configuration as shown in (c).

Experimental and Computational Methods

Experimental Methods

IRAS and XPS experiments were conducted in a UHV system for surface reactivity studies(1) using a $TiO_2(110)$ ($5 \times 5 \times 0.5$ mm^3) single crystal from CrysTec GmbH. The samples were mounted with Ta clips onto a Ta backplate, using a thin Au sheet in between to improve thermal contact. The sample was cooled using a liquid He flow cryostat and heated to the desired temperature by resistively heating the Ta backplate. The vacuum chamber is equipped with a home-built effusive molecular beam source, where the gas streams out of an orifice with an effective diameter of 38.0 ± 1.9 μm. This source delivers an almost ideal top-hat profile at the sample with a 3.5 mm diameter and beam core pressure of $3.0 \pm 0.3 \times 10^{-8}$ mbar at the sample position.(2) The base pressure in the chamber was below 10^{-10} mbar. Monochromatized Al K_α irradiation from an Al/Ag twin anode X-ray source (Specs XR50 M, FOCUS 500) and a hemispherical analyzer (Specs Phoibos 150 with a delayline detector) were used for XPS measurements. The energy scale is calibrated after each bakeout using copper, silver, and gold foils attached to the cryostat. The IRAS spectra were recorded using our newly developed setup for measuring dielectrics.(3) The $[001]$ surface vector of the $TiO_2(110)$ sample is in the plane of incidence. This implies that for p-polarized light, the electric field component parallel to the surface oscillates predominantly in the direction of the Ti and O rows, and that of s-polarized light is perpendicular to the rows. The p signal is more pronounced in our design compared to other setups due to the selected non-grazing angular range for p-polarized light on $TiO_2(110)$. The range of incidence angles was set to 48°–65° for p-polarized light (i.e., the non-grazing side of the Brewster angle). The IRAS spectra in this work originate from the difference of the

sample reflectivity spectrum R and that of the adsorbate-free surface, R_0. The normalized reflectivity difference is defined as

$$\frac{\Delta R}{R_0} = \frac{R - R_0}{R_0} .$$

The sample was cleaned by cycles of sputtering (15 min, 1 keV Ne^+, $I_{sample} \approx 1$ µA/cm²) and annealing (900 K, 15 min). As a final step, the sample was first oxidized by annealing in O_2 (5×10^{-7} mbar O_2, 900 K, 30 min) to avoid overreduction, and then vacuum-annealed (900 K, 15 min).(4,5) XPS scans after the cleaning procedure confirmed the absence of impurities on the surface within the detection limit. The density of oxygen vacancies was ≈12% with respect to the number of surface unit cells, as judged from D_2O TPD spectra. Rh was deposited using a water-cooled e-beam evaporator (FOCUS EFM3), whose flux was calibrated using a temperature-stabilized quartz microbalance (QCM). One monolayer (ML) corresponds to 1 Rh atom per surface unit cell.

Scanning probe microscopy (SPM) was done in a separate UHV system consisting of an analysis chamber with a base pressure lower than 10^{-11} mbar and a preparation chamber with a base pressure lower than 10^{-10} mbar. The sample was prepared in the same way as for the XPS and IRAS measurements, with annealing temperatures of ≈1100 K. The O defect density amounted to ≈11% of all surface unit cells as determined by STM. The images were obtained using a qPlus sensor(6) (f_0 = 31.8 kHz, k = 1800 N/m, $Q \approx 10000$) with an electrochemically etched tungsten tip. Typical oscillation amplitudes were A = 150 pm. The base temperature in the SPM head was 4.7 K, but the sample was counter-heated to 14 K in order to provide sufficient conductivity for STM, unless otherwise noted. STM was measured with a positive sample bias, tunneling into the empty states of the sample (U = +0.5 V to +3.0 V). An atomically sharp metal tip was prepared on a Cu(110) single crystal by voltage pulses

and dipping the tip into the surface. Images were processed by correcting for the piezo creep and drift(7) and filtering a few frequencies of mechanical noise in the Fourier domain. Rh was deposited using an e-beam evaporator (EFM3, FOCUS) cooled with liquid N_2, with the flux calibrated using a temperature-stabilized QCM, and 1 ML corresponding to 1 Rh atom per surface unit cell.

Computational methods

All calculations were performed using the Vienna *ab initio* simulation package (VASP).(8) The projector augmented wave (PAW) method(9,10) was employed for the near-core regions and the plane-wave basis set cutoff energy was set to 700 eV. To more accurately capture the electronic structure around the Rh atom, we used 17 active electrons, and accordingly the VASP PAW potentials O_h, C_h, Rh_sv_GW and Ti_sv. Calculations were performed using optPBE-vdW, a spin-polarized GGA method. This approach integrates the optPBE exchange-correlation functional(11) with non-local correlation corrections from vdW-DF as proposed by Dion et al., effectively incorporating van der Waals interactions.(12,13) An effective on-site Coulomb repulsion term U_{eff} = 3.9 eV was applied to the *d*-orbitals of the Ti atoms.(14) The unreconstructed rutile TiO_2(110) surface was modeled as an asymmetric slab comprising five TiO_2 tri-layers within a large two-dimensional 6 × 2 unit cell and including a vacuum region greater than 12 Å along the z-axis. The top three tri-layers were allowed to relax, while the bottom two tri-layers were kept fixed at their bulk positions. Additionally, pseudo-hydrogen atoms were used to saturate the bottom layer to fulfill the octet rule; this makes the bottom surface more bulk-like and thereby allows using thinner slabs.(15) The convergence criterion was an electronic energy step of 10^{-7} eV and forces acting on ions smaller than 0.01 eV/Å.

The adsorption energies were computed according to the formula

$$E_{ads} = \left(E_{Rh/TiO_2 + nCO} - \left(E_{Rh/TiO_2} + nE_{CO}\right)\right)/n$$

where $E_{Rh/TiO_2 + nCO}$ is the total energy of the slab with the Rh adatom on TiO_2(110) with *n* adsorbed CO molecules, E_{Rh/TiO_2} is the total energy of the slab with the adsorbed Rh adatom on the TiO_2(110) surface, and E_{CO} represents the energy of the CO molecule in the gas phase. The Rh 3*d* core-level binding energies were calculated using the initial state approximation.(16,17) Atomic charges were determined through Bader charge analysis.(18) The CO vibrational frequencies were computed within the harmonic approximation, employing the finite-difference method.

For improved accuracy in frequency calculations, we used the HSE06 hybrid functional(19) with the standard mixing factor 25% and a screening length of 0.2^{-1} $Å^{-1}$. The geometry was fully reoptimized accordingly, and all HSE06 calculations were performed using a plane-wave basis set cutoff energy of 400 eV with standard PBE pseudopotentials. The convergence criteria were set to an electronic energy threshold of 10^{-6} eV and ionic forces below 0.01 eV/Å. The computed CO stretching vibrational frequency for adsorbed CO on the Rh/TiO_2(110) was scaled by the method-dependent factor $v_{CO_{gas}}^{exp}/v_{CO_{gas}}^{cal}$,with $v_{CO_{gas}}^{exp}$= 2143 cm^{-1}, and $v_{CO_{gas}}^{cal}$= 2114 cm^{-1} and 2232 cm^{-1} for optPBE-vdW and HSE06, respectively.

1. Pavelec J, Hulva J, Halwidl D, Bliem R, Gamba O, Jakub Z, et al. A multi-technique study of CO_2 adsorption on Fe_3O_4 magnetite. *J. Chem. Phys.* 2017, 146(1).

2. Halwidl D. Development of an Effusive Molecular Beam Apparatus. Wiesbaden: Springer Fachmedien Wiesbaden; 2016.

3. Rath D, Mikerásek V, Wang C, Eder M, Schmid M, Diebold U, et al. Infrared reflection absorption spectroscopy setup with incidence angle selection for surfaces of non-metals. *Rev. Sci. Instrum.* 2024, 95(6).

4. Diebold U. The surface science of titanium dioxide. *Surf. Sci. Rep.* 2003, 48(5–8):53–229.

5. Wendt S, Schaub R, Matthiesen J, Vestergaard EK, Wahlström E, Rasmussen MD, et al. Oxygen vacancies on TiO_2(110) and their interaction with H_2O and O_2: A combined high-resolution STM and DFT study. *Surf. Sci.* 2005, 598(1–3):226–45.

6. Giessibl FJ. The qPlus sensor, a powerful core for the atomic force microscope. *Rev. Sci. Instrum.* 2019, 90(1).

7. Choi JIJ, Mayr-Schmölzer W, Mittendorfer F, Redinger J, Diebold U, Schmid M. The growth of ultra-thin zirconia films on Pd_3Zr(0001). *J. Phys.: Condens. Matter* 2014, 26(22):225003.

8. Kresse G, Furthmüller J. Efficiency of ab-initio total energy calculations for metals and semiconductors using a plane-wave basis set. *Comput. Mater. Sci.* 1996, 6(1):15–50.

9. Blöchl PE. Projector augmented-wave method. *Phys. Rev. B* 1994, 50(24):17953–79.

10. Kresse G, Joubert D. From ultrasoft pseudopotentials to the projector augmented-wave method. *Phys. Rev. B* 1999, 59(3):1758–75.

11. Dion M, Rydberg H, Schröder E, Langreth DC, Lundqvist BI. Van der Waals Density Functional for General Geometries. *Phys. Rev. Lett.* 2004, 92(24):246401.

12. Klimeš J, Bowler DR, Michaelides A. Chemical accuracy for the van der Waals density functional. *J. Phys.: Condens. Matter* 2010, 22(2):022201.

13. Klimeš J, Bowler DR, Michaelides A. Van der Waals density functionals applied to solids. *Phys. Rev. B* 2011, 83(19):195131.

14. W Wang Z, Brock C, Matt A, Bevan KH. Implications of the DFT+U method on polaron properties in energy materials. *Phys. Rev. B* 2017, 96(12):125150.

15. Kowalski PM, Meyer B, Marx D. Composition, structure, and stability of the rutile TiO_2(110) surface: Oxygen depletion, hydroxylation, hydrogen migration, and water adsorption. *Phys. Rev. B* 2009, 79(11):115410.

16. Lizzit S, Baraldi A, Groso A, Reuter K, Ganduglia-Pirovano MV, Stampfl C, et al. Surface core-level shifts of clean and oxygen-covered Ru(0001). *Phys. Rev. B* 2001, 63(20):205419.

17. Köhler L, Kresse G. Density functional study of CO on Rh(111). *Phys. Rev. B* 2004, 70(16):165405.

18. Henkelman G, Arnaldsson A, Jónsson H. A fast and robust algorithm for Bader decomposition of charge density. *Comput. Mater. Sci.* 2006, 36(3):354–60.

19. Krukau AV, Vydrov OA, Izmaylov AF, Scuseria GE. Influence of the exchange screening parameter on the performance of screened hybrid functionals. *J. Chem. Phys.* 2006, 125(22).
